# Facile synthesis of the Ti^3+^ self-doped TiO_2_-graphene nanosheet composites with enhanced photocatalysis

**DOI:** 10.1038/srep08591

**Published:** 2015-02-26

**Authors:** Bocheng Qiu, Yi Zhou, Yunfei Ma, Xiaolong Yang, Weiqin Sheng, Mingyang Xing, Jinlong Zhang

**Affiliations:** 1 Key Lab for Advanced Materials and Institute of Fine Chemicals, East China University of Science and Technology, Shanghai. 200237, China

## Abstract

This study developed a facile approach for preparing Ti^3+^ self-doped TiO_2_-graphene photocatalyst by a one-step vacuum activation technology involved a relative lower temperature, which could be activated by the visible light owing to the synergistic effect among Ti^3+^ doping, some new intersurface bonds generation and graphene oxide reduction. Compared with the traditional methods, the vacuum activation involves a low temperature and low-costing, which can achieve the reduction of GO, the self doping of Ti^3+^ in TiO_2_ and the loading of TiO_2_ nanoparticles on GR surface at the same time. These resulting TiO_2_-graphene composites show the high photodegradation rate of MO, high hydrogen evolution activity and excellent IPCE in the visible light irradiation. The facile vacuum activation method can provide an effective and practical approach to improve the performance of TiO_2_-graphene and other metal oxides-graphene towards their practical photocatalytic applications.

Nanoscaled TiO_2_ photocatalyst has attracted much more attention due to its stable chemical and physical property, lower cost and excellent photocatalytic performance^1^,^2^,^3^,^4^. However, the broad bandgap of TiO_2_ seriously restrains its practical application in the area of full-wavelength of solar spectrum. Recently, graphene (GR) is used as an ideal supporter to load with TiO_2_ nanoparticles, due to the excellent conductive properties, mechanical properties and chemical stability^5^,^6^,^7^,^8^,^9^. Although the TiO_2_-graphene has become a new photocatalyst with efficient photoactivity, there are also some problems on this novel photocatalytic composites, such as the origin of its visible light response^10^,^11^,^12^. And another problem is the choosing of the appropriate reductant using for the reduction of GO. Hydrazine hydrate is widely used as the reductant in application of the reduction of GO^13^,^14^, but it has high-toxicity and is easy to cause the secondary pollution^15^,^16^. As a nontoxic reductant, alcohols are also usually used to reduce the GO^6^,^14^,^17^. Its reducing capacity is relatively weak, which can only reduce the epoxy groups in GO. The hydroxy and carboxyl groups are difficult to be reduced by the alcohols. Actually, the GR obtained by the alcohol reduction is the intermediate and transition state, whose structure and property are falling in between the GO and the entirely reduced GR. Most importantly, the wet chemical reduction method can easily introduce some impurities. Hence, it is necessary to develop a clean technology for the reduction of GO, which becomes a research hotspot on the studying of graphene-semiconductor composites.

Herein, we developed a one-step vacuum activation method to achieve the reduction of GO, the self doping of Ti^3+^ in TiO_2_ and the loading of TiO_2_ nanoparticles on GR surface at the same time. The GO and the commercial P25 were mixed together and activated in vacuum at 300°C for 3 hours. The obtained composites were denoted as “V-P25/nGR” (“V” indicates the vacuum activation; “n” indicates the volume of GO, n = 0.02, 0.05, 0.10, 0.50, 1.0 mL). For comparison, The pure P25 heated in vacuum without the addition of GO was denoted as V-P25. The vacuum activated GR and vacuum activated P25 were mixed together by the mechanical mixing method, which was denoted as the “M-P25/0.10GR” (“M” indicates the mechanical mixing process). H-P25/0.5GR (“H” indicates the hydrothermal process) was prepared through a hydrothermal method.

## Results

**Figure 1:**
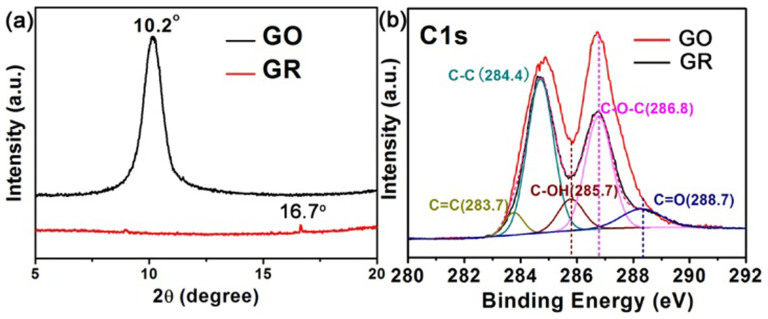
XRD patterns and XPS analysis of samples. XRD (a) patterns and C1s XPS (b) spectra for the GO before and after vacuum activation. The C1s XPS spectra of GO and GR are consistent with our previous work^9^,^23^.

The earthy yellow GO was heated at 300°C in vacuum for 3 h to obtain the black reduced GR, and its TEM spectrum in Figure S1 indicated that the GR still keeps the large two-dimensional structure after the vacuum activation. XRD patterns and C1s XPS spectra are given to demonstrate the reduction of GO in the one-step vacuum activation process. After heated in vacuum, the characteristic peak for GO shifts from 10.2° to 16.7° (Figure 1a), which indicates the reduction of GO to obtain GR. The peak at a small angle of 16.7° is indicative of GR with lower crystallinity and some defects^18^. Except the introducing impurity of C-C (284.4 eV), there are major four fitting peaks in the C1s XPS spectrum of GO (Figure 1b). The peaks at 283.7, 285.7, 286.8 and 288.7 eV are ascribed to the skeleton C = C, the surface C-OH, the surface epoxy group of C-O-C and the surface carboxyl group of C = O respectively^9^,^19^,^20^,^21^,^22^. After treatment of the vacuum activation, the peaks of C-OH, C-O-C and C = O on GO have a distinct decrease, resulting from the reduction of GO in the vacuum heating process. This result is consistent with our previous work^9^,^23^. Different from the wet reduction process involving the reductants of hydrazine hydrate and ethanol, vacuum activation is a nontoxic and highly reduction degree technology, which can reduce the surface hydroxyl group, epoxy group and carboxyl group, simultaneously.

**Figure 2:**
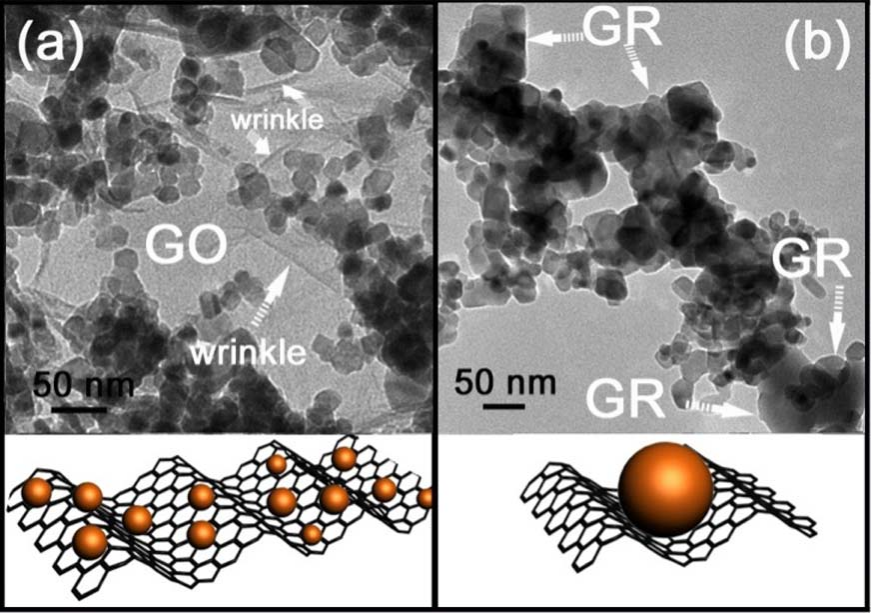
TEM images for different samples. TEM spectra for the V-P25/0.10GR before (a) and after (b) vacuum activation.

**Figure 3:**
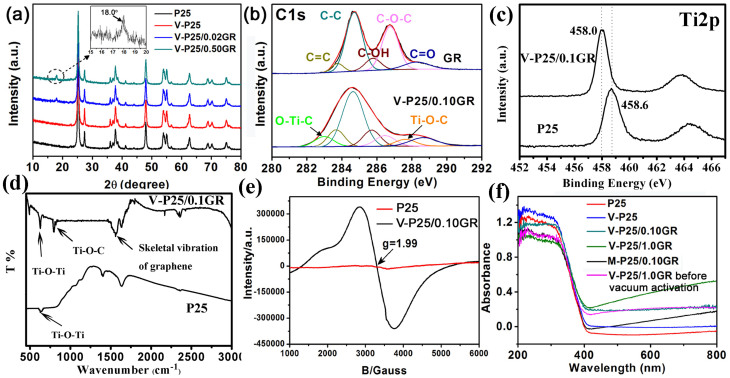
XRD, XPS, FTIR, EPR and UV-DRS analysis of different samples. XRD patterns for the TiO_2_-Graphene composites (a); C1s XPS spectra for the GR with and without P25 loading (b); Ti2p XPS spectra for pure P25 and V-P25/0.10GR (c); FTIR spectra of pure P25 and V-P25/0.10GR (d); EPR spectra for the composite after vacuum activation (e); UV-DRS spectra for the TiO_2_-Graphene composites (f).

When nano-sized P25 was mixed with GO and heated in vacuum for 3 h, its surface morphology exhibited a significant change, as shown in the TEM spectra of Figure 2. The large two-dimensional structure of GO is cut into a small size of GR with lamellar structure. Compared with the morphology of blank GR (Figure S1b), the decrease of dimensions of GR after loading with P25 indicates that the interaction between TiO_2_ nanoparticles and GR not only can promote the transportation and separation efficiency of photoexcited charge carriers, but also cut the large GR into small pieces to form the nanosheets. Before vacuum activation treatment, the TiO_2_ are loaded on the surface of large sheet of GO (Figure 2a), however, after the vacuum activation, the nanoparticles are composited with some smaller sized GR sheets (Figure 2b). In order to overcome the localised analysis of TEM characteriztion, we randomly chose a location to observe the mophology of V-P25/0.10GR, as shown in Figure S1c,d. There are some obvious smaller sized GR sheets existence in the composite of V-P25/0.10GR after vacuum activation, which indicates the "cutting" of large GO sheets into smaller size during the vacuum activation treatment. In order to investigate the interaction between TiO_2_ and GR, some other characterizations are also provided. The G band in the Raman spectrum of V-P25/0.10GR exhibits a distinct decrease from 1610.7 to 1598.7 cm^−1^, indicating the reduction of GO to GR in the vacuum activation process (Figure S2). The obvious increase of the intensity ratio of D/G bands after vacuum activation treatment confirms the reduction of GO and the cutting of large GO sheets into smaller sized GR sheets (from 0.91 to 1.10). The smaller GR sheets should contain higher density of edge carbon (associated with the defect D band), which induces the increase of the intensity ratio of D/G bands. The characteristic peaks of TiO_2_ in XRD patterns of V-P25/nGR are still changeless (Figure 3a), which suggests the mild process of vacuum activation technology with few effects on the crystal form of P25. It is a remarkable fact that the peak assigned to GR is changing from 16.7° to 18.0° after loading with TiO_2_ nanoparticles. The shift may be resulted from the existence of chemical connection between TiO_2_ nanoparticles and GR surface carbon groups. In our previous work, we have found that the macro-residual stress induced by the foreign impurity of TiO_2_ nanoparticles can give a macro-residual stress to the GR and cause the anisotropic lattice contraction^9^, which is the reason for the cutting of large GR into small pieces. When the macro-residual stress is the pressure stress, the diffraction peaks will shift to the high angle, whereas the tensile stresses will make the diffraction peaks shift to the low angle. The loading of TiO_2_ on GR surface exhibits a distinct pressure stress, which leads to the characteristic peak of GR changing from 16.7° to 18.0°. The C1s XPS spectra also indicate the existence of chemical binding between TiO_2_ and GR (Figure 3b). Compared with the blank GR, the two new peaks at 282.5 and 285.5 eV are ascribed to the O-Ti-C and Ti-O-C respectively^9^,^24^,^25^,^26^, accompanying with a relative decrease of characteristic peak of C-O-C after loading with the P25 nanoparticles. In addition, the Ti2p XPS spectra (Figure 3c) shows that the Ti2p_3/2_ peak of V-P25/0.1GR shifts from 458.6 eV to a lower binding energy owing to the increasement of the electron density of Ti atom in TiO_2_, suggesting the existence of Ti-O-C bonds on the surface of TiO_2_. Moreover, Figure 3d shows the FIRT spectra of the V-P25/0.1GR and P25, respectively. For V-P25/0.1GR, the small peak around 628 cm^−1^ is assigned to the vibration of Ti-O-Ti bonds and the peak at 798 cm^−1^ is corresponded to the the vibration of Ti-O-C bonds. Besides, the peak around 1726 cm^−1^ is assigned to C = O stretching of the residual COOH groups. However, pure P25 only shows a small peak at 628 cm^−1^ at low frequency (below 1000 cm^−1^), which is corresponded to the vibration of Ti-O-Ti bonds. The above results further confirm the existence of Ti-O-C bonds and the reduction of GO. Different with our previous work for the preparation of the composite of P25/boron doped GR by a stirring-ultraphonic method^9^, the P25/GR prepared by the vacuum activation is beneficial to the formation of O-Ti-C bonds between P25 and GR, which are expected to narrow the bandgap of TiO_2_. It can be deduce that the –OH on TiO_2_ surface is reacting with the C-O-C groups on GO surface to form the Ti-O-C and O-Ti-C bonds in the vacuum activation process. These new bonds will introduce a distinct pressure stress on GR surface to divide the large scaled GR sheet into smaller pieces, as shown in Figure 2b. Much more exposed edges on GR can give a fringe effect and facilitate the further modification on the edge of GR^27^,^28^. Hence, the interaction force between GR and TiO_2_ is a chemical interaction, which can divide the large scaled GR into small pieces and provide a new approach to synthesize GR nanosheet. This chemical interaction also can supply a transmission electron bridge between TiO_2_ and GR.

In addition to the reduction of GO and the generation of new bonds at the interface of TiO_2_ and GR, the Ti^3+^ self-doping modification on TiO_2_ also can be achieved at the same time by the one-step vacuum activation method. Our previous reports have confirmed that the low temperature vacuum activation method is an effective technology to produce stable active Ti^3+^ in TiO_2_ bulk, which could enhance the visible light absorption of TiO_2_^29^. The EPR spectrum for V-P25/0.10GR shows a very strong signal at g = 1.99, indicating the generation of Ti^3+^, while no signal is observed for the pure P25 (Figure 3e). The Ti^3+^ is responsible for the enhancing absorption of V-P25 in the visible light region^29^,^30^, as shown in Figure 3f. The composites of Ti^3+^ self-doped TiO_2_ loaded with GR exhibit a strong red shift of absorption band. Compared with vacuum activated composites, the M-P25/0.10GR prepared by the mechanical mixing method only shows an obvious visible light absorption, but its absorption band does not change too much. There is also changeless for the absorption band of V-P25/1.0GR before vacuum activation, indicating that the vacuum heating process plays an important role in the narrowing of bandgap of TiO_2_. Kubelka-Munk function is used as the vertical axis and plotted against photon energy, as shown in Figure S3. The corresponding band energies of different composites obtained from Figure S3 are shown in Table 1. A little decrease band energy of V-P25 from 3.03 eV to 3.01 eV is induced by the formation of Ti^3+^ local state inside the TiO_2_ band gap. After P25 loading on GR surface through a vacuum activation method, its band gap has been reduced to 2.96 eV, which results from the formation of some surface shallow defect states in TiO_2_^25^. Several groups have reported that various composite synthesis techniques result in the formation of Ti-O-C and O-Ti-C bonds which cause a red-shift of the TiO_2_ absorption band edge^31^,^32^,^33^. Ti-O-C and O-Ti-C bonds have the same effect on TiO_2_ as impurities: they introduce defect states into the TiO_2_ band gap, allowing the photogeneration of electrons from lower-energy photons. However, the band energy of M-P25/0.10GR being similar with the pure P25 suggests the mechanical mixing is only a physical process, which is difficult to give an obvious reduction of the bandgap of TiO_2_.

## Discussion

**Figure 4:**
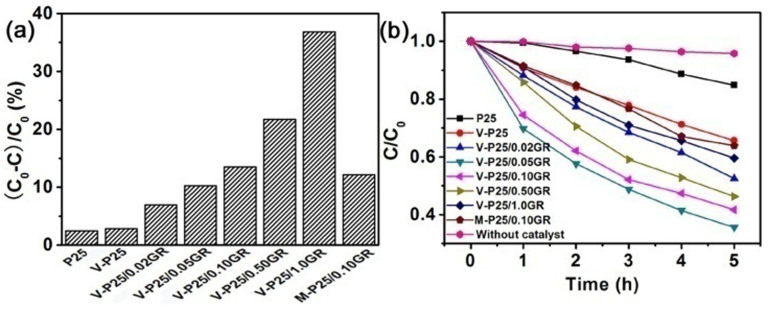
Photocatalytic activities of different samples. Adsorption capacities of MO on different samples (a); Visible light photocatalytic activities of different samples (b).

All the TiO_2_-graphene composites show an excellent adsorption capacity of MO in the dark because of the π-π conjugation accumulation effect between GR and dyes (Figure 4a)^34^. And the adsorption rate is increasing with the increase of GR added. However, with the increase of GR addition, the photodegradation rate of MO over P25-graphene composites in the visible light irradiation increased first and then decreases obviously (Figure 4b). When the GR addition amount is 0.05 mL, the corresponding catalyst has the optimal photocatalytic activity. Analogous activity trend for photodegradation of methylene blue (MB), is also observed over the P25-GR nanocomposites in some other previous reports^6^,^34^. The catalyst of M-P25/0.10GR prepared by the mechanical mixing method also has a high adsorption capacity, but its photocatalytic activity is much lower than that of V-P25/0.10GR. This result indicates that the interaction between TiO_2_ and GR plays an important role in the enhancing of visible light photoactivity.

**Figure 5:**
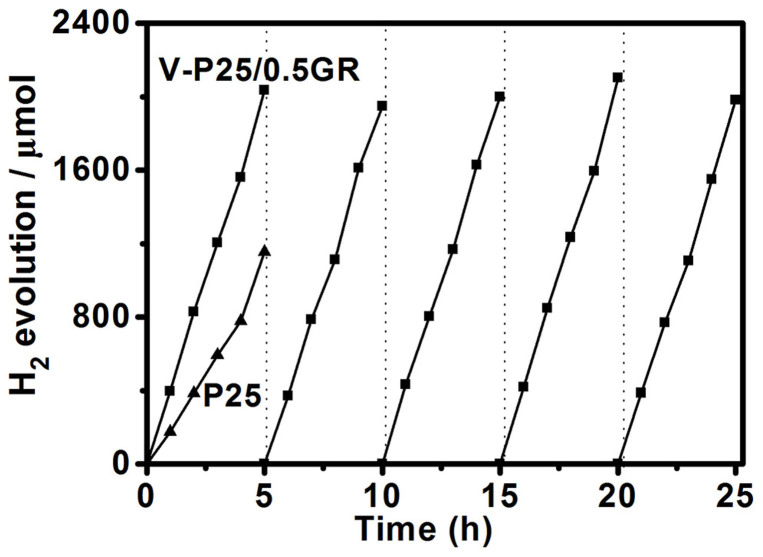
Photocatalytic H_2_ evolution. Photocatalytic H_2_ evolution performance and stability test on V-P25/0.5GR and P25 under visible light irradiation (λ > 400 nm).

We also studied the photocatalytic activity of P25 and V-P25/0.5GR nanocomposites in hydrogen evolution, using Pt as a co-catalyst under visible light irradiation (λ > 400 nm) (Figure 5). P25 exhibits a moderate hydrogen evolution rate of 200 μmol·h^−1^, whereas V-P25/0.5GR nanocomposite shows an obviously increased hydrogen evolution rate of 400 μmol·h^−1^, which is around two times higher than that of P25. Moreover, the production of H_2_ increased steadily with irradiation time, without inactivation after five cycles. These experiments results indeed prove the stability of the structure and reactivity of V-P25/GR. Meanwhile, these results indicate the potential application and promising prospect of V-P25/GR composites in photocatalytic devices.

**Figure 6:**
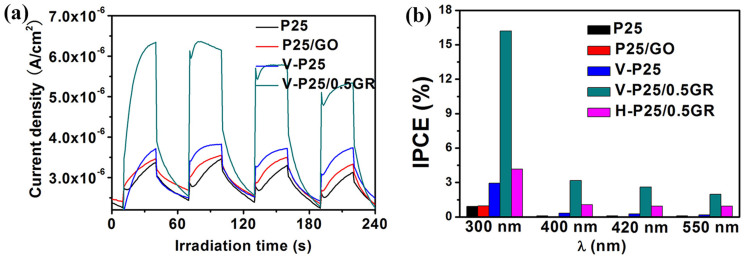
Photoelectrochemical Tests. Transient photocurrent responses for different samples under the simulated solar light irradiation (a); Incident photon to current conversion efficiency (IPCE) of different samples under different wavelength illumination (b).

Next, we have used the photoelectrochemistry to characterize the lifetime and transfer rate of photogenerated electron–hole pairs. The transient photocurrent response measurements exhibits the photocurrent of V-P25/0.5GR is much higher than that of P25, P25/GO or V-P25 (Figure 6a), testifying the low efficiency of recombination of photogenerated electron–hole pairs in the V-P25/0.5GR, that is, vacuum activation process and graphene addition can greatly promote the separation efficiency of photogenerated electron–hole pairs. We have also noticed that the IPCE in the UV-visible light region for V-P25/0.5GR is significantly higher than that of other samples (Figure 6b). Even under the illumination at the wavelength of 550 nm, the IPCE for V-P25/0.5GR still remains at the value of ~2. The catalyst of H-P25/0.5GR prepared by the hydrothermal method presents a lower IPCE than V-P25/0.5GR. That is, compared with the hydrothermal method, it is well established that more Ti^3+^-dopant through a vacuum activation process leads to better visible light absorbance. This result is also consistent with the result of photoactivity. Meanwhile, it deserves to be mentioned that the value of IPCE for V-P25/0.5GR is up to ~16.5% under the illumination of at the wavelength of 300 nm. This outstanding IPCE of V-P25/0.5GR is ascribed to the reduction of GO, the self-doping of Ti^3+^, and the loading of TiO_2_ nanoparticles on GR surface. The excellent IPCE of V-P25/0.5GR indicates its large application potential in solar cell.

**Figure 7:**
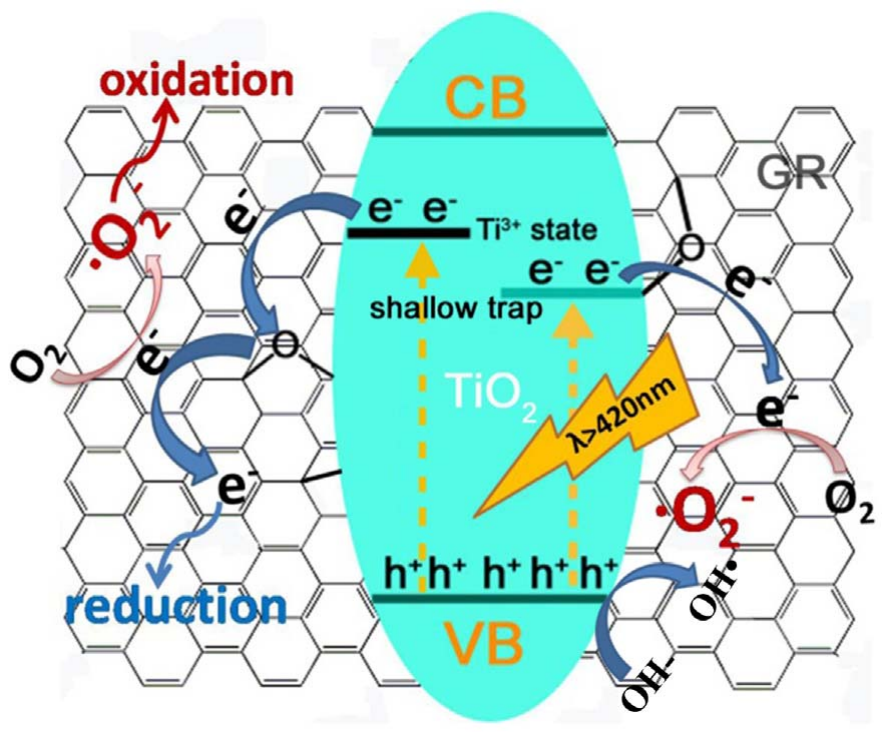
Schematic representation of the Photocatalytic reaction. Band structure model for Ti^3+^ self-doped TiO_2_-Graphene composite and schematic diagram of photo-excited electrons transfer among Ti^3+^ doped TiO_2_ and GR sheets.

In the one-step vacuum activation process, there are major three modifications occurring at one time: 1) the reduction of GO to GR; 2) the formation of Ti-O-C and O-Ti-C structures; 3) the self-doping of Ti^3+^. The produced Ti^3+^ could introduce some local states inside the band gap of TiO_2_^29^,^35^,^36^, as shown in Figure 7. These states will narrow the bandgap of TiO_2_ and benefit to the separation of photo-generated electrons and holes, which is the reason for the higher photoactivity of V-P25. The Ti-O-C and O-Ti-C structures could introduce some shallow trap states at the surface or intersurface of TiO_2_^26^,^33^, and further narrow the TiO_2_ bandgap from 3.03 eV to 2.96 eV (Table 1). This providential decrease of bandgap can increase the light absorption of TiO_2_/GR composite in the visible light region without significant decreasing the reducing power of electrons in TiO_2_ and GR sheets. In addition, the introduction of shallow trap states can improve the transfer efficiency of electrons between TiO_2_ and GR, which will enhance the life-times of photogenerated carriers. Thereby, the V-P25/0.5GR has an enhanced hydrogen evolution capacity in the visible light irradiation. The chemical bonds also can provide a transmission bridge to efficiently transfer the electrons from TiO_2_ to GR. The reduction of GO could promote the electrical conductivity of GR, and improve the transfer efficiency of photo-electrons from TiO_2_ to GR. The electrons on GR surface will produce a large number of active species such as ·O_2_^−^ to degrade the dye pollutants. Certainly, the electrons on GR also can directly split water to produce H_2_ under the visible light irradiation. Seen from the Figure 4a, the MO molecules prefer to be adsorbed on the surface of GR sheets, which means the active species on the surface of GR play an important role in the degradation of MO under the visible light irradiation. On the other hand, we cannot completely eliminate the dye photosensitization in the photodegradation of MO. Under the visible light irradiation, the adsorbed MO molecules also can generate the electrons and transfer them to the GR sheets, which will increase the number of active species of ·O_2_^−^ to degrade the dye pollutants. Simultaneously, in the photo-degradation of MO, the absence of sacrificial hole scavenger promotes the collective holes on TiO_2_ VB reacting with H_2_O molecules to generate many mobile free OH radicals (Figure 7). A large number of generated mobile free OH radicals are also beneficial to the photocatalytic activity of V-P25/GR for the degradation of MO under the visible light irradiation.

The above mentioned synergistic effect among Ti^3+^ doping, new bonds generation and the reduction of GO is the reason for the enhanced photocatalytic activity of V-P25/nGR. To the sample of M-P25/0.10GR, the decrease of Ti-O-C and O-Ti-C bonds makes the photo-excited electrons only localized on the TiO_2_ surface, which cannot transfer from TiO_2_ to GR. The lower transfer efficiency of electrons leads to the lower photocatalytic activity of M-P25/0.10GR, which makes the photodegradation rate much lower than V-P25/0.10GR (Figure 4b).

In conclusion, a one-step vacuum activation method was exploited to successfully prepare the Ti^3+^ self-doped TiO_2_-graphene composites. The reduction of GO, the self-doping of Ti^3+^, and the loading of TiO_2_ nanoparticles on GR surface were achieved at the same time. The loading of TiO_2_ nanoparticles could produce a pressure stress on GR surface to divide the large scaled GR sheet into small pieces, which could expose much more edges to give a fringe effect and facilitate the further modification on the edges. The synergistic effect among Ti^3+^ doping, new interface bonds generation and GO reduction are responsible for the high photodegradation rate of MO and high hydrogen evolution rate in the visible light irradiation, which supplied an economic and powerful way in environmental cleaning. The high photocatalytic activity and IPCE of V-P25/nGR suggest its large application potential in energy development and solar cell.

## Methods

### Preparation of GO and blank GR

Graphene oxide (GO) was synthesized from natural graphite powder using a modified Hummers methods^37^. The detail experimental procedures were referring to the report published by Zhangpeng Li et al^38^. 5 ml GO was dispersed into 20 ml double distilled water and ultraphonic for 1 hour, and then the solution was drying under 60°C for 12 h. Obtained brown flaky solid was heated at 300°C for 3 hours in the vacuum condition. The color of flaky solid was changing from brown to black. This black solid was dispersed into 20 ml double distilled water again and ultraphonic for 10 hours. The solid products were washed by the double distilled water for 5 times and dispersed into 5 ml double distilled water. Finally, it was transferred to a plastic bottle and denoted as GR.

### Synthesis of TiO_2_-graphene composites

A certain amount of GO solution (2.0 mg/ml) was added into 40 ml double distilled water and ultraphonic for 1 hour, and then 0.5 g P25 (Degussa) was added into the solution. The mixture solution was ultraphonic for 1 hour and vigorous stirring for another 1 hour. The mixture was dried under 80°C and heated in the vacuum at 300°C for 3 hours. The obtained composites were denoted as “V-P25/nGR” (“V” indicates the vacuum activation; “n” indicates the volume of GO, n = 0.02, 0.05, 0.10, 0.50, 1.0 ml). The pure P25 heated in the vacuum without the addition of GO was denoted as V-P25. The vacuum activated GR (0.10 ml) and vacuum activated P25 (0.5 g) was mixed together by the mechanical mixing method, which was denoted as the “M-P25/GR” (“M” indicates the mechanical mixing process). H-P25/0.5GR (“H” indicates the hydrothermal process) was prepared through a hydrothermal method. In detail, 0.5 mL GO (2.0 mg/ml) was added into 40 ml double distilled water and ultraphonic for 1 hour, and then 0.5 g P25 (Degussa) was added into the solution. The mixture solution was ultraphonic for 1 hour, then transferred into 100 mL Teflon-lined stainless-steel autoclave and heated at 180°C for 12 h. After cooling naturally, the as-prepared sample was dried at 60°C.

### Characterization

X-ray diffraction (XRD) patterns of all samples were collected in the range 10–80° (2θ) using a RigakuD/MAX 2550 diffractometer (Cu K radiation, λ = 1.5406 Å), operated at 40 kV and 100 mA. The morphologies were characterized by transmission electron microscopy (TEM, JEM2000EX). The instrument employed for X-ray photoelectron spectroscopy (XPS) studies was a Perkin-Elmer PHI 5000C ESCA system with Al Kα radiation operated at 250 W. The shift of the binding energy due to relative surface charging was corrected using the C1s level at 284.4 eV as an internal standard. The X-band electron paramagnetic resonance (EPR) spectra were recorded at room temperature (Varian E-112).Raman measurements were performed at room temperature using a ViaReflex Raman spectrometer with the excitation wavelength of 514 nm. The UV-vis absorbance spectra were obtained for the dry-pressed disk samples using a Scan UV-vis spectrophotometer (Varian, Cary 500) equipped with an integrating sphere assembly, using BaSO_4_ as the reflectance sample. The spectra were recorded at room temperature in air within the range 200–800 nm.

### Photoelectrochemical Tests

Incident photon to electron conversion efficiency (IPCE) were measured under the illumination of a 300 W xenon lamp by an AM 1.5 G solar simulator with a monochromator (Newport), given by IPCE = 100% × 1240/λ × Y/Y_ref_ × Resp_det_. Where λ is the wavelength of light (nm), Y is the photocurrent (A cm^−2^) under illumination at λ, and Y_ref_ represents the reference scan photocurrent. The reference detector Y_ref_ has the responsivity Resp_det_. Photocurrent measurements were carried out with an analyzer (Zahner, Zennium) in dark conditions using a standard three electrode cell with a working electrode, a Pt wire as the counter electrode, and a saturated calomel electrode as the reference electrode. A 0.5 M solution of Na_2_SO_4_ was used as the electrolyte.

### Photocatalytic degradation of organic pollutants

Visible light-driven photocatalytic activity of each sample was evaluated in terms of the degradation of methyl orange (MO, 10 mg/L). The photocatalyst (0.07 g) was added into a 100 mL quartz photoreactor containing 70 mL of an organic pollution solution. The mixture was stirred for 120 min in the dark in order to reach the adsorption–desorption equilibrium. A 500-W tungsten halogen lamp equipped with a UV cut-off filters (λ > 420 nm) was used as a visible light source. At the given time intervals, the analytical samples were taken from the mixture and immediately centrifuged, then filtered through a 0.22 μm Millipore filter to remove the photocatalysts. The filtrates were analyzed by recording variations in the absorption in UV-vis spectra of MO using a Cary 100 ultraviolet visible spectrometer.

### Photocatalytic hydrogen evolution

Photocatalytic hydrogen evolution experiments were performed in a Pyrex top-irradiation reaction vessel which was connected to a closed gas circulation and a vacuum system. A 300 W top-irradiated Xenon lamp with a UV–cutoff filter (λ > 400 nm) was used as a light source. Typically, 0.1 g of the catalyst was suspended in a 100 ml methanol solution (20 vol%). 1 mL H_2_PtCl_6_·6H_2_O aqueous solution was added into the system to load 0.37 wt% Pt onto the surface of the photocatalyst by a in situ photodeposition method under irradiation. Prior to illumination, the system was always deaerated for 15 min by evacuation in order to remove the dissolved air from the system under constant stirring. The generated hydrogen was analyzed by gas chromatograph (GC7890, Tian Mei, Shanghai, TCD, nitrogen as a carrier gas and 5 Å molecular sieve column).

## Author Contributions

M.X. and J.Z. conceived and designed the experiments. M.X. and B.Q. prepared the samples and performed characterization. B.Q., Y.Z., Y.M., X.Y., W.S., M.X. and J.Z. were mainly responsible for preparing the manuscript. All the authors discussed the results and reviewed the manuscript.

## Supplementary Material

Supplementary Information Supporting Information

## Figures and Tables

**Table 1:** The band energies of different samples

Samples	P25	V-P25	V-P25/1.0GR	M-P25/0.10GR
Band Energy (eV)	3.03	3.01	2.96	3.04
