# Supplementary material for: Facile synthesis of the Ti3+ self-doped TiO2-graphene nanosheet composites with enhanced photocatalysis
Source: Sci Rep. 2015 Feb 26;5:8591. doi: 10.1038/srep08591 (PMC4341197; doi:10.1038/srep08591)
Supplement: Supplementary Information — Supporting Information [file srep08591-s1.doc]

Supporting information

**Facile synthesis of the Ti^3+^ self-doped TiO_2_-Graphene nanosheet composites with enhanced photocatalysis**

Bocheng Qiu, Yi Zhou, Yunfei Ma, Xiaolong Yang, Weiqin Sheng, Mingyang Xing* and Jinlong Zhang*

Key Laboratory for Advanced Materials and Institute of Fine Chemicals, East China University of Science and Technology, 130 Mei long Road, Shanghai 200237, P.R.China

* E-mail: mingyangxing@ecust.edu.cn; jlzhang@ecust.edu.cn


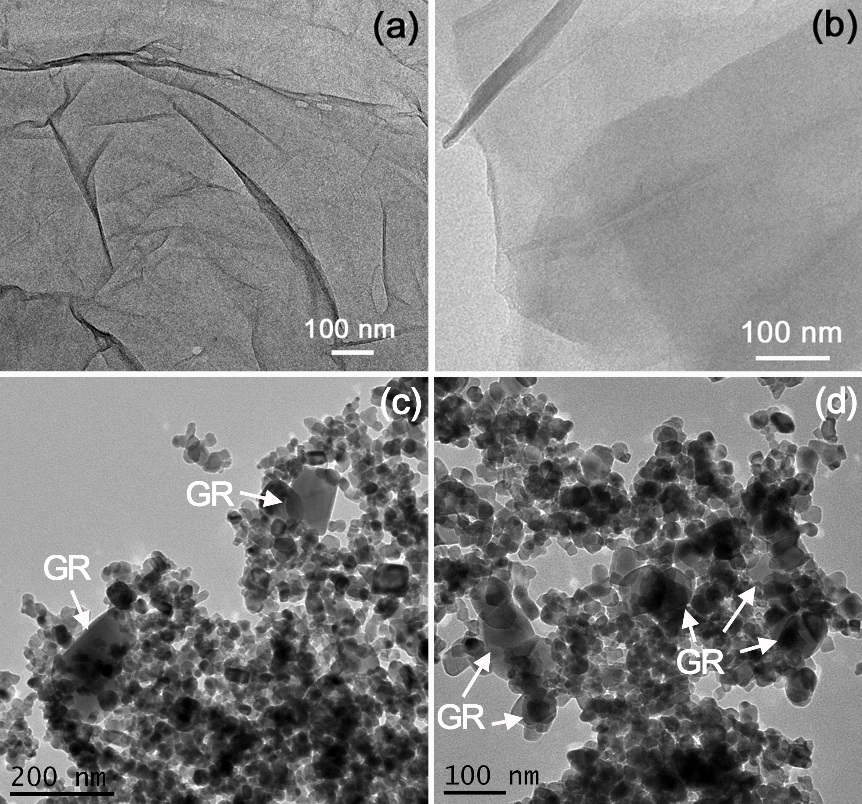


**Figure S1.** TEM images for the GO (a) and GR (b). (c, d) TEM images for the V-P25/0.10GR after vacuum activation.


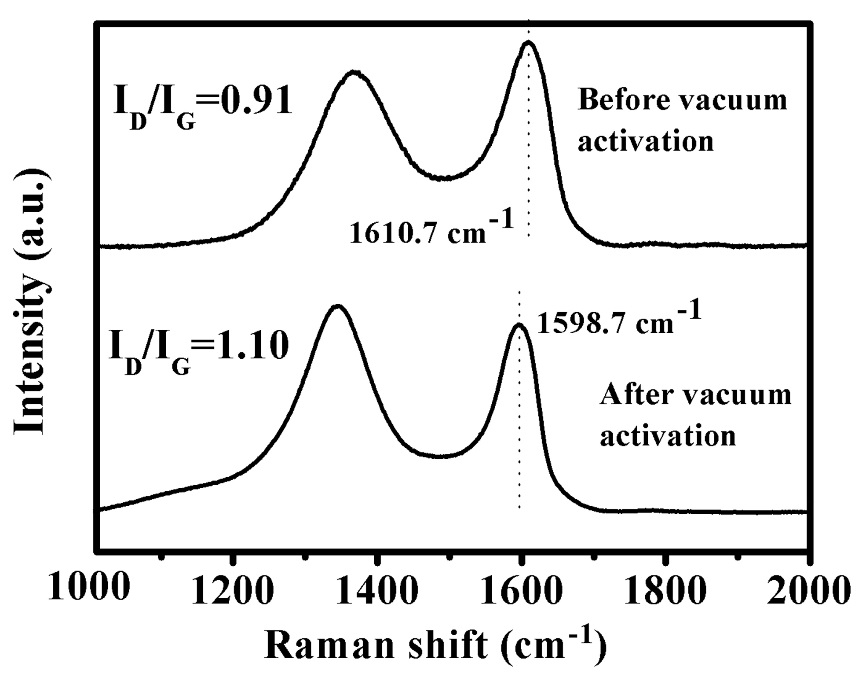


**Figure S2.** Raman spectra for V-P25/0.10GR before and after vacuum activation.


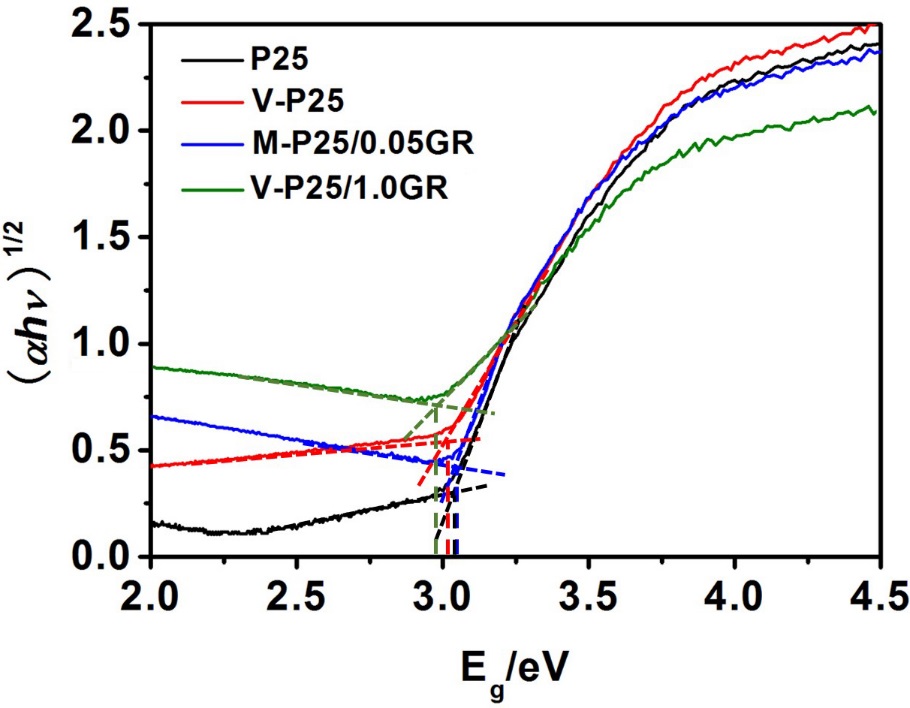


**Figure S3.** Curve of Kubelka-Munk function plotted against the photon energy over different samples
